# Cannabis use patterns in drug-resistant and pharmacoresponsive epilepsy: Single tertiary referral center survey investigation

**DOI:** 10.1371/journal.pone.0281040

**Published:** 2023-01-27

**Authors:** Danielle McDermott, Marielle L. Darwin, Kirsten Fetrow, Ian Coulter, Kristal Biesecker, John A. Thompson

**Affiliations:** 1 Department of Neurology, School of Medicine, University of Colorado Anschutz Medical Campus, Aurora, CO, United States of America; 2 Department of Neurosurgery, School of Medicine, University of Colorado Anschutz Medical Campus, Aurora, CO, United States of America; 3 School of Medicine, University of Colorado Anschutz Medical Campus, Aurora, CO, United States of America; University of Catania, ITALY

## Abstract

This study sought to identify differences in cannabis use and perceptions about cannabis in mitigating seizure-related symptoms in patients with epilepsy, and to evaluate differences in these patterns between drug-resistant versus pharmacoresponsive epilepsy. A collection of self-report surveys completed by patients with epilepsy (*n* = 76) were used to retrospectively compare differences in those with drug-resistant versus pharmacoresponsive epilepsy regarding 1) proportion who used cannabis, 2) frequency of use, 3) method of use, and 4) reason for use. A Cochran-Armitage test for trend indicated that of patients who used cannabis, a higher proportion of patients in the drug-resistant group used more frequently than in the pharmacoresponsive group. Almost half (48%) of those in the drug-resistant group reported daily use compared to approximately a third (36%) of those in the pharmacoresponsive group. Additionally, no patient in either group reported that cannabis was harmful in relation to seizure-related symptoms. Results from this study highlight the need for epilepsy providers to formally assess patients’ perceptions and use of non-prescribed cannabis to inform clinical care decisions, particularly in the drug-resistant epilepsy population.

## Introduction

Epilepsy is a chronic, highly prevalent neurological disease, affecting 3.4 million Americans with 150,000 new cases diagnosed annually [1,2]. Antiseizure medications (ASMs) are the mainstay treatment effective in approximately 65% of people with epilepsy, leaving the remaining 35% resistant to medications [3]. Poorly managed seizure control can impact physical and psychiatric health as well as employment and educational opportunities, especially for patients with drug resistant epilepsy [4].

Prior work has explored the use of cannabis as a treatment option for epilepsy [5,6]. Federal policy in the United States and the classification of cannabis as a schedule 1 drug constrains systematic investigation of medicinal properties of cannabis [7]. Recent state-based legalization of cannabis for designated medical criteria arose from mounting scientific evidence in potential medicinal benefits, followed by legalization for recreational purposes through citizen-driven ballot-based initiatives in 18 states and the District of Columbia [7]. In addition, large scale randomized clinical trials provide Class I evidence for the efficacy of cannabidiol (CBD), a cannabinoid compound naturally secreted by the Cannabis sativa L. plant, in treatment of specific epilepsy syndromes. For instance, CBD was approved by the Federal Drug Administration (FDA) in 2018 to treat seizures associated with Lennox–Gastaut and Dravet syndrome [8] as well as those associated with tuberous sclerosis complex [9].

Access to pharmaceutical CBD is limited to patients with specific approved indications. As a result, many patients with epilepsy use alternative, commercially available CBD-based supplements and tetrahydrocannabinol (THC; the primary psychoactive component in cannabis) products, commonly referred to as artisanal cannabis. However, these CBD products are less refined and commonly contain THC in combination with varying concentrations of CBD [10]. Despite their widespread availability, the evaluation of the safety and efficacy of these products is limited, and a determination of their clinical utility carries inherent uncertainty and potential risk.

Moreover, studies suggest that in recent decades, cannabis use has been rising while the perceived risk of use has been declining [7,11]. The increase in use, acceptability, and expansion of controlled clinical trials of cannabis are key influencers in patient and provider interest in medicinal applications of cannabis. In the backdrop of expanded access to artisanal cannabis, and the need for the continued development of controlled clinical trials, it is imperative for patients, clinicians, and investigators to understand beliefs and perceptional influences that may impact use, safety, and efficacy of prescription cannabis. The development of safe and efficacious cannabis-based treatments hinges on attaining a better understanding of patient use patterns and potential differences in those with drug resistant and pharmacoresponsive epilepsy. This information will provide insight into patients’ needs and advance communication when evaluating therapeutic options for people with epilepsy, and if these needs differ for drug resistant versus pharmacoresponsive patients.

The primary aim of the current study was to identify differences in cannabis use and perceptions regarding the usefulness of cannabis in mitigating seizure-related symptoms in patients’ epilepsy in a tertiary referral center. Secondary aims were to evaluate differences in these patterns between patients with drug resistant versus pharmacoresponsive epilepsy. Due to pharmacological failure in those with drug resistant epilepsy, it was hypothesized that patients in the drug resistant epilepsy group would have higher rates and frequency of use, may be more inclined to perceive that cannabis affects seizure symptoms, and perceive cannabis to be beneficial for seizure-related symptoms compared to those in the pharmacoresponsive epilepsy group. The methods of use and the specific symptoms that cannabis was perceived to help were additionally compared between groups, though these analyses were exploratory in that no supposition was put forth regarding the relative behavioral differences between patients with drug resistant and pharmacoresponsive epilepsy. By meeting the study aims of examining the differences between these two groups, clinicians and clinical trial researchers will gain a better understanding of the factors that relate to cannabis use in these sub-populations to inform treatment.

## Material and methods

### Participants

Eligible participants were identified by the study team at the Comprehensive Epilepsy Center at University of Colorado Hospital and included male and female adults (age ≥21) with a definitive clinical diagnosis of epilepsy confirmed by a neurologist (see **Table 1** for sample characteristics). The type of epilepsy reported in Table 1 was verified by the patients’ charts. The study was approved by Colorado Multi-Institutional Review Board (COMIRB) at the University of Colorado. Informed consent was obtained electronically in written format. Of the 83 eligible participants, 76 agreed to enroll in the study. Participants were volunteers and received no compensation for completing the study.

**Table 1:** Study sample descriptive characteristics.

	Drug resistant (*n* = 52)	Pharmacoresponsive (*n* = 24)
Age	*M* = 40.1 ± 11.6 [22–74]	*M* = 39.3 ± 11.4 [27–69]
Race and ethnicity (%) Hispanic or Latinx White Black or African American Asian Other Prefer not to answer	7.7 82.6 1.9 1.9 0.0 5.9	8.3 75.0 4.2 4.2 1.9 6.4
Biological sex (%) Female Male Prefer not to answer	61.5 36.6 1.9	87.5 12.5 0.0
Duration of diagnosis (years, mode)	16–20	6–10
Epilepsy type (%) Focal Generalized Unknown	94 2 4	67 17 17
History of surgery (%)	71	4
Of patients with surgery–Multiple surgeries (%)	32	0

We have considered potential differences in cannabis use in Colorado compared to other states due to differing legal statuses. Previous studies suggest that there is no significant difference in use rates when comparing states based on the legal availability of cannabis [7]. While cannabis use in legalized states is generally higher than non-legalized states, these trends were present before legalization occurred. It appears that a rise in cannabis use at a state level is reliably associated with legalization of cannabis in that state [7]. Due to these findings, we consider our results to be generalizable to other samples in different states.

### Study design and procedure

Patients completed a collection of surveys that were developed with consultative input from a collaborative team within our campus including specialists in addiction medicine and drug use behaviors [12]. The surveys were subsequently pilot tested among a patient focus group. The surveys were completed independently by use of a browser-based electronic platform (Qualtrics Software, Provo, UT) [13] during their regular clinic appointment. Data collection occurred over the course of eight months from July 2020 to February 2021.

Designation of drug resistant epilepsy was assigned based on retrospective review of medical records using International League Against Epilepsy (ILAE) criteria, specifically defined as the “failure of adequate trials of two tolerated and appropriately chosen and used ASM schedules (whether as monotherapies or in combination) to achieve sustained seizure freedom. [14] Information regarding cannabis use patterns and cannabis-related measures were obtained from the self-report survey questions.

### Data analysis

All data was processed and analyzed using the statistical software R (R Core Team, 2020) [15]. These analyses aimed to examine the differences in cannabis use patterns in patients with drug resistant (n = 52) vs. pharmacoresponsive (n = 24) epilepsy. Specifically, differences between these two groups were explored regarding 1) proportion who used cannabis, 2) frequency of use, 3) method of use, and 4) reason for use.

### Use of cannabis

Dichotomous answers to the survey question, “Have you used cannabis in the past 12 months?” were used in a Fisher’s exact test to determine if the proportion of cannabis users differed between people with drug resistant vs. pharmacoresponsive epilepsy.

### Frequency of cannabis use

Answers to the survey question, “In the past 12 months, how often have you used cannabis?” informed patients’ frequency of use. The answer choices were presented as a Likert scale ranging from 1 to 4 (1 = Once a month or less; 2 = More than once a month; 3 = More than once per week; 4 = Daily). A Cochran-Armitage test for trend determined if in those with drug resistant epilepsy, there was a greater proportion of individuals who used cannabis at higher frequencies compared to those with pharmacoresponsive epilepsy.

### Method(s) of cannabis use

Patients were asked to indicate what their preferred method(s) of cannabis use were by selecting all methods that applied out of the following answer choices: Joint/cigarette, electronic cigarette/vape pen, edible, liquid extract (oil), transdermal (lotion, ointment, cream), concentrate (dab/wax), other [open-ended text description]. The examination of methods of use and use with multiple modalities between those with drug resistant vs. pharmacoresponsive epilepsy were exploratory and the differences between groups were considered without the use of statistical tests.

### Reported effects of cannabis use

Patients were asked a series of questions to assess if and how they believe that cannabis affected their preictal, ictal or interictal seizure-related symptoms, and if they experienced any general negative side effects from cannabis use (i.e., negative effects not necessarily related to epilepsy). A Fisher’s exact test determined if answers to the question, “Do you believe using cannabis affects your seizure symptoms?” differed between the drug resistant and pharmacoresponsive groups. To examine the perceived benefit of cannabis use, patients were asked “How beneficial is cannabis on your seizure-related symptoms?” The answer choices to the latter were presented as a Likert scale ranging from 1 to 5 (1 = Absolutely beneficial; 2 = Some benefit; 3 = No effect; 4 = Some harm; 5 = Absolutely harmful). A Wilcoxon Rank-Sum test determined if there were differences between groups. In addition, information was collected on the reported side effects of cannabis using additional survey questions. First, the patients were asked, “Have you ever experienced negative side-effects from cannabis?” If the response was “yes”, participants were asked “Have negative side effects caused you to stop using cannabis?” and to select the top three side effects that they experienced from a provided list with an option to write in additional reasons.

Lastly, patients indicated what symptoms they believed that cannabis helped by selecting all that applied out of the following answer choices: Lowers seizure frequency, Lowers intensity of a seizure, Reduces drowsiness after a seizure, Reduces headache after a seizure, Improves sore muscles after a seizure, Faster recovery, Cannabis helps with no symptoms, Other [open-ended text description]. The differences between drug resistant vs. pharmacoresponsive epilepsy were exploratory and were considered without the use of statistical tests.

## Results

### Use of cannabis

**Fig 1:**
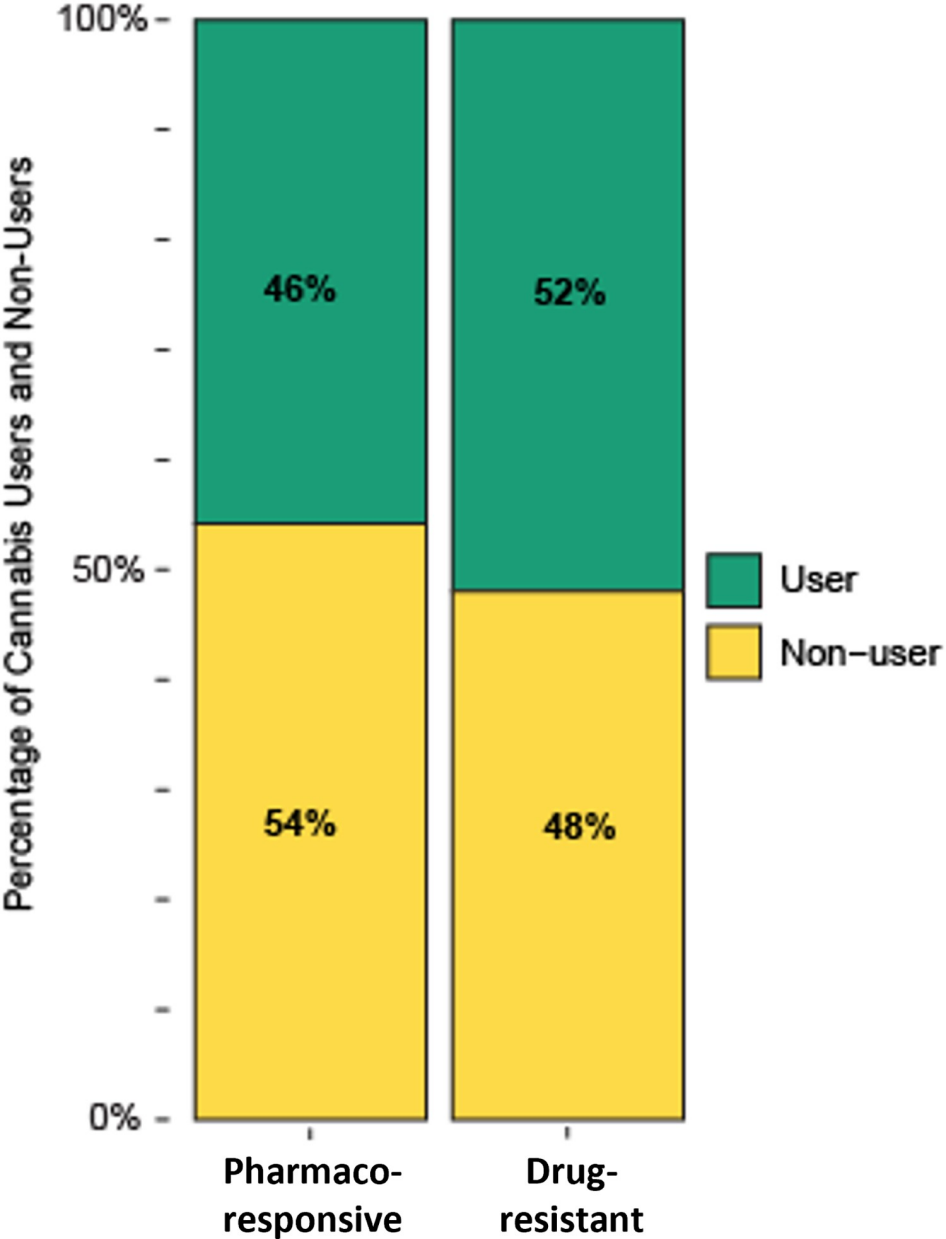
Proportion of cannabis users in study sample. Results of Fisher’s exact test and relative distributions of cannabis use in participants with drug-resistant and pharmacoresponsive epilepsy.

Overall, 38 of 76 (47%) patients surveyed reported cannabis use in the last 12 months. Within this sample, 27 of 52 (52%) patients with drug resistant epilepsy and 11 of 24 (46%) patients with pharmacoresponsive epilepsy reported cannabis use. There was no statistically significant difference in cannabis use between the two groups (p = .805, CI [0.264 2.303] (Fig 1).

### Frequency of cannabis use

**Fig 2:**
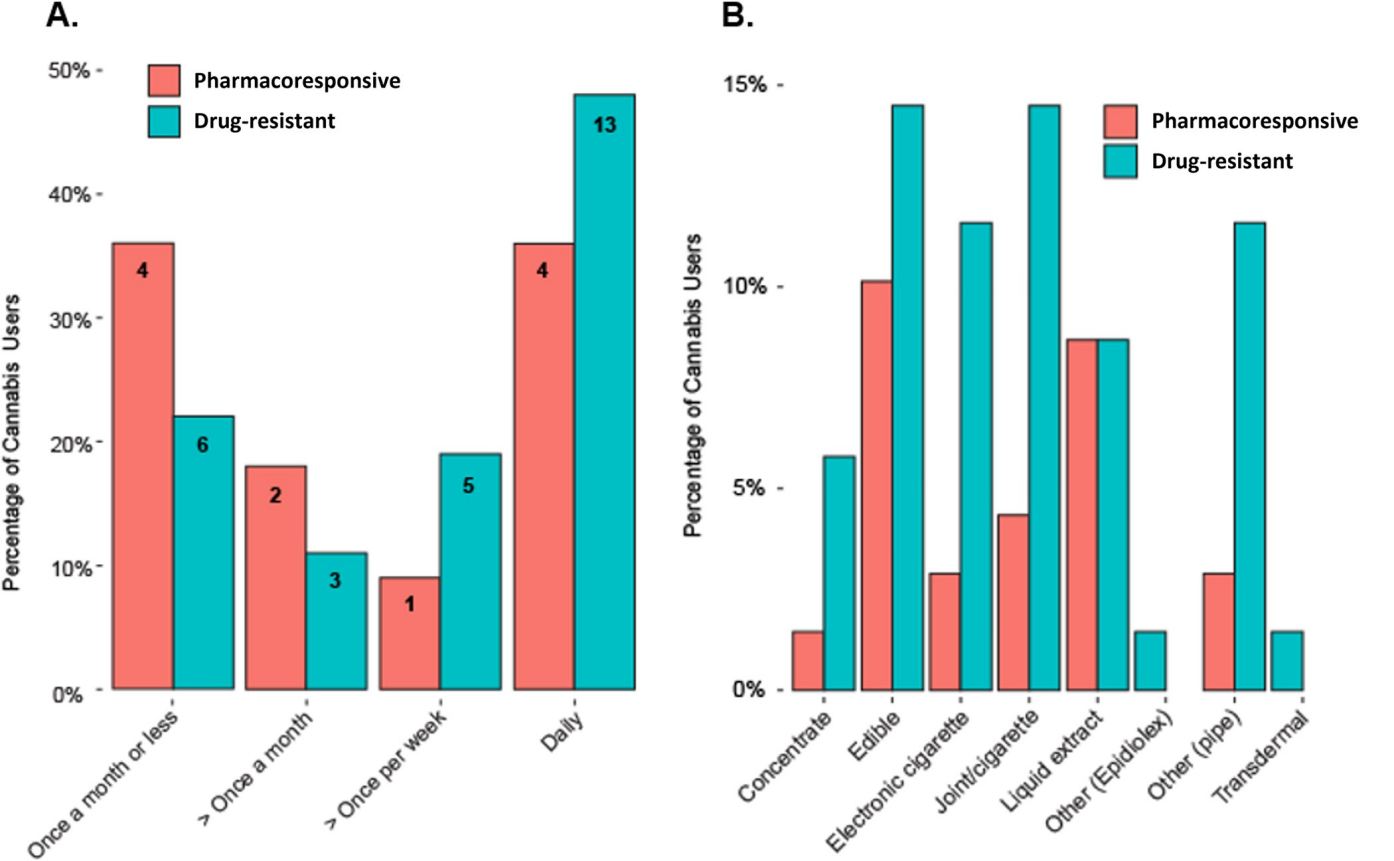
Frequency and method of cannabis use. 2a. Among those who use cannabis, there was a higher proportion of more frequent users in patients with drug-resistant vs. pharmacoresponsive epilepsy. The numbers inside the bars represent the number of patients in each group who used cannabis at a specific frequency. 2b. Comparison of percent of method(s) of cannabis use in participants with drug-resistant and pharmacoresponsive epilepsy.

Of those who used cannabis in the drug resistant group (n = 27), 6 (22%) reported use once a month or less, 3 (11%) more than once a month, 5 (19%) more than once a week, and 13 (48%) reported daily use. Of those who used cannabis in the pharmacoresponsive group (n = 11), 4 (36%) used once a month or less, 2 (18%) used more than once a month, 1 (9%) used more than once a week, and 4 (36%) reported daily use. A Cochran-Armitage test for trend indicated that of patients who used cannabis, a statistically higher proportion of patients in the drug resistant group used more frequently than in the pharmacoresponsive group (Z = -2.6189, p = 0.004) (Fig 2A).

### Method(s) of cannabis use

An exploratory analysis examined the method(s) of cannabis use and the presence of multiple modalities of use amongst those with drug resistant epilepsy compared to those with pharmacoresponsive epilepsy. Participants were able to select more than one method of use when completing the survey. Results indicate that the most frequent methods of use in the drug resistant group were joints/cigarettes (14.5%) and edibles (14.5%), while the most frequent methods of use in the pharmacoresponsive group were edibles (10.1%) and liquid extracts (8.7%). The least frequent methods of use were comparable between the drug resistant and pharmacoresponsive groups, being other, Epidiolex ® (1.4% and 0%, respectively) and transdermal applications (1.4% and 0%, respectively) (Fig 2B). The proportion of users in each group who used cannabis by multiple modalities was evaluated as well. In the drug resistant group (n = 27), 14 (52%) used with a single modality, 9 (33%) used two different modalities, 2 (7%) used three different modalities, and 2 (7%) used four different modalities. In the pharmacoresponsive group (n = 10), 3 (30%) used with a single modality, 5 (50%) used two different modalities, 2 (20%) used three different modalities, and none used four different modalities.

### Reported effects of cannabis use

**Fig 3:**
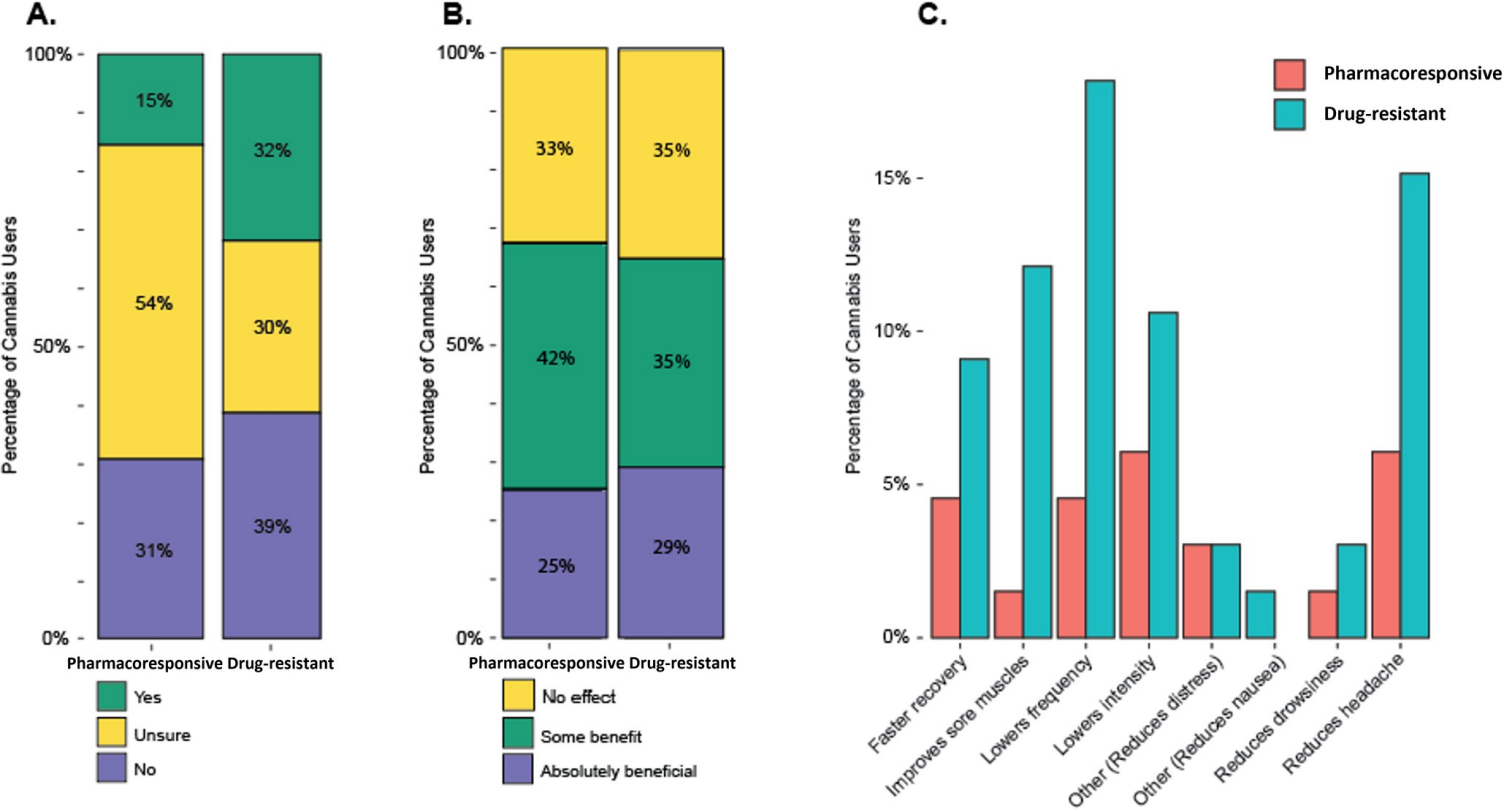
Reasons for cannabis use. 3a. Belief that cannabis affected epilepsy symptoms in patients with drug-resistant vs. pharmacoresponsive epilepsy. 3b. Perceived benefit of cannabis on seizure-related symptoms in patients with drug-resistant vs. pharmacoresponsive epilepsy. 3c. Differences in seizure-related symptoms that cannabis was perceived to help in patients with drug-resistant vs. pharmacoresponsive epilepsy.

A series of analyses probed the question if and how people with drug resistant vs. pharmacoresponsive epilepsy believe that cannabis affected their seizure symptoms. A comparison of answers to the question, “Do you believe using cannabis affects your seizure symptoms?” yielded that of patients with pharmacoresponsive epilepsy, 15% endorsed that cannabis affected seizure symptoms, 31% did not believe so, and the remaining 54% were unsure. Of those in the drug resistant group, 32% stated that they believed cannabis affected symptoms, 39% believed not, and the remaining 30% were unsure. Although the data was in the hypothesized direction, a Fisher’s exact test determined that there was no statistically significant difference between the two groups (p = .297) (Fig 3A).

Patients were asked "How beneficial is cannabis on your seizure-related symptoms?" to which the answer choices were presented as a Likert scale ranging from 1 to 5 (1 = Absolutely beneficial; 2 = Some benefit; 3 = No effect; 4 = Some harm; 5 = Absolutely harmful). 67% of those with pharmacoresponsive epilepsy and 64% of those with drug resistant epilepsy stated that cannabis was either “Absolutely beneficial” or provided “Some benefit”, while the remaining patients stated that cannabis had no effect on their seizure-related symptoms. No patient in either group endorsed that cannabis was harmful in relation to their seizure-related symptoms. A Wilcoxon Rank-Sum test determined that there were no statistically significant differences in responses between groups (W = 188, p = 0.966) (Fig 3B). Although no patient indicated that cannabis negatively affected seizure-related symptoms, 18 of the 76 patients (24%) reported that they had experienced general negative side effects from cannabis use, and of those 18, 8 patients (44%) indicated that negative side effects caused them to stop using cannabis. The top three negative side effects endorsed from the full sample (*n* = 76) were: Difficulty concentrating (*n* = 10, 13%), memory problems (*n* = 7, 9%), and increased fatigue (*n* = 6, 8%).

Lastly, patients indicated what symptoms they believed cannabis helped by selecting all that applied out of the provided answer choices with opportunity to write in any responses. The most frequently endorsed seizure-related effects alleviated by cannabis use were lowering the intensity of seizures (6.1%) and reducing headaches after seizures (6.1%) in the pharmacoresponsive group, while lowering seizure frequency (18.2%) and lowering the intensity of seizures (10.6%) were most endorsed in the drug resistant group (Fig 3C). Although the types of symptoms that cannabis helped differed, the number of symptoms that cannabis helped was comparable between groups with a mean of 2.45 symptoms for the drug resistant group and a mean of 2.22 symptoms for the pharmacoresponsive group.

## Discussion

This study is the first to examine differences in cannabis use patterns in those with drug resistant versus those with pharmacoresponsive epilepsy. There is increased use of cannabis as a treatment option for epilepsy, including FDA approved medications such as Epidiolex® (GW Research Ltd, Cambridgeshire, United Kingdom). The increase in accessibility, acceptability, and controlled study of cannabis are key circumstances that are promising for exploring cannabis as a treatment. Future development is further predicated on understanding patients’ perceptions of the treatment effect. With this factor in mind, clinicians may need to formally determine their patients’ use and perception of cannabis-based treatments to optimize or ensure safe treatment decisions. Distinguishing the use patterns and beliefs about use that differ between those with drug resistant and pharmacoresponsive epilepsy are important to understand to best target cannabis-based treatments and understand symptom management. With the knowledge that a significant proportion of individuals with epilepsy use non-prescribed cannabis, and that negative patient perceptions of use regarding help with seizure-related symptoms are minimal, we see potential for prescription use to increase.

Our findings indicate that there was a significantly higher proportion of more frequent users in the drug resistant group compared to the pharmacoresponsive group (*p* = 0.004). Almost half (48%) of those in the drug resistant group reported daily use compared to approximately a third (36%) of those in the pharmacoresponsive group. Patients with drug resistant epilepsy are at higher risk of lower quality of life compared to those with pharmacoresponsive epilepsy due to higher perceived stigma, diminished cognitive well-being, psychopathologies such as anxiety and depression, less independence, and higher medical costs [16]. The higher proportion of more frequent cannabis users in the drug resistant group could reflect a more present need to manage non-seizure aspects. Future studies should investigate this inference by more directly examining the relationship between the frequency of cannabis use and quality of life changes in those with pharmacoresponsive and drug resistant epilepsy.”

Further results indicate that the majority (67% in pharmacoresponsive and 64% in drug resistant group) of the study sample found cannabis to be beneficial for seizure-related symptoms. Although there was no statistically significant difference between groups regarding perceived benefit, the higher proportion of frequency of use found in the drug resistant group may be a result from widely perceived benefits of cannabis use. Patients in the drug resistant group reported that cannabis use helped with a mean of 2.45 seizure-related symptoms, a clinically significant amount that may improve quality of life for those with drug resistant epilepsy. The higher proportion of daily users in the drug resistant group compared to the pharmacoresponsive group may help to guide clinicians’ recommendations for cannabis-related treatment options.

Additionally, when asked how beneficial cannabis was on seizure-related symptoms, no patient in either group reported that cannabis was harmful in relation to seizure-related symptoms. Patients in both groups reported that cannabis either had no effect (33% in pharmacoresponsive and 35% in drug resistant group) or was beneficial (67% in pharmacoresponsive and 64% in drug resistant group) towards seizure-related symptoms. Further research is needed to consider the utility of cannabis-based treatments of comparable efficacy to first line ASMs before conclusions can be made. In addition, it may be the case that the perception of cannabis’ helpfulness to reduce symptoms varies with certain ASMs. While we ultimately chose not to pursue this line of investigation as we did not assess whether the patients’ seizure-related symptoms were directly caused by ASMs or not, such an inquiry would be informative to explore in subsequent studies.

Lastly, it is important to consider the differing levels of seizure frequencies between the drug resistant and pharmacoresponsive groups when interpreting these results. Since those in the pharmacoresponsive group have experienced no seizures in the past four weeks, seizure-related symptoms can be expected to be lower and thus follows that the reported effects of cannabis on seizure-related symptoms can be expected to be lower as well.

### Limitations

There are a number of limitations to the current study that ought to be considered with the interpretations of our findings. Our surveys did not make the distinction between patients who used cannabis recreationally (e.g., to get high, to fit into social situations) those who used for therapeutic purposes (e.g., to improve appetite, induce sleep, relieve nausea, relieve pain), or those who were motivated to use for multiple purposes. Determining the primary intention of patients’ use of cannabis would expand our understanding of the reasons for use that the patients reported in this study. It is unknown if those who reported a positive effect on symptoms were using recreationally and that the effect on seizure-related symptoms was inadvertent. In addition, the current study did not assess the amount of cannabis consumed. This facet would help to further identify key differences in user profiles and may supplement the findings related to perceived effects on seizure-related symptoms. Subsequent studies would benefit from including these inquires in their investigations. Third, conclusions regarding the differences in cannabis use patterns between pharmacoresponsive and pharmacoresistant patients are limited without a larger sample. The results of the study intend to be exploratory in nature to provide direction for future studies to continue this line of investigation.

### Clinical relevance and future directions

The beneficial elements of cannabis use are critical to identify to fully understand the mechanisms in which cannabis aids people with epilepsy. Cannabis may be an effective AED on its own, it may improve efficacy of existing AEDs, and it may increase quality of life-related factors related to seizure activity if not seizure activity itself. Our study aimed to explore this latter supposition by gaining a better understanding of cannabis use patterns in this population and how use related to perceived differences in quality of life. The findings that cannabis was perceived to be beneficial in relation to seizure-related symptoms and that a higher proportion of those with drug resistant epilepsy used cannabis more frequently are key circumstances that are promising for treatment when bearing in mind the increase in use, acceptability, and controlled study of cannabis outside of this study sample. However, future development must be preceded by investigations of additional factors that may affect the patients’ willingness to use the treatment. With this in mind, clinicians may wish to target cannabis-based treatments in patients who use cannabis already, identify those with positive believes about use, or explore challenging negative beliefs about use. Distinguishing these use patterns and beliefs about use that differ between those with drug resistant and pharmacoresponsive epilepsy are important to understand to best target cannabis-based treatments and understand symptom management.
